# Dietary ω-3 intake for the treatment of morning headache: A randomized controlled trial

**DOI:** 10.3389/fneur.2022.987958

**Published:** 2022-09-20

**Authors:** Marco Marchetti, Paola Gualtieri, Antonino De Lorenzo, Domenico Trombetta, Antonella Smeriglio, Mariarosaria Ingegneri, Rossella Cianci, Giulia Frank, Giulia Schifano, Giulia Bigioni, Laura Di Renzo

**Affiliations:** ^1^PhD School of Applied Medical-Surgical Sciences, University of Rome Tor Vergata, Rome, Italy; ^2^Section of Clinical Nutrition and Nutrigenomics, Department of Biomedicine and Prevention, University of Tor Vergata, Rome, Italy; ^3^Department of Chemical, Biological, Pharmaceutical and Environmental Sciences (ChiBioFarAm), University of Messina, Messina, Italy; ^4^Dipartimento di Medicina e Chirurgia Traslazionale, Università Cattolica del Sacro Cuore, Fondazione Policlinico Universitario “Agostino Gemelli” IRCCS, Rome, Italy; ^5^School of Specialization in Food Science, University of Tor Vergata, Rome, Italy; ^6^Department of Physics, University of Rome Sapienza, Rome, Italy

**Keywords:** morning headache, migraine, ω-3 fatty acids, eating habits, lifestyle, Mediterranean Diet

## Abstract

Morning headache (MH) is a frequent condition with a not fully explained pathogenesis. During the past years, several studies have been performed to identify a better diet therapy to reduce the frequency and intensity of headaches. Our study aims to determine if an adequate omega-3 (ω-3) intake in a Modified Mediterranean Diet (MMD) can improve the frequency and intensity of MH and, subsequently, the quality of life. Of the 150 enrolled subjects, 95 met the inclusion criteria (63.3%). Patients were included in the study and randomized into two groups: group A following MMD A and group B following MMD B. The MMD A group was designed to arise at least a 1.5:1 ω-6/ω-3 ratio; the MMD B group was designed to derive at least a 4:1 ω-6/ω-3 ratio. Eighty-four subjects completed the 6 weeks intervention (56%). After 6 weeks (t1), group A showed a significant reduction in both Headache Impact Test-6 and Visual Analog Scale scores (*p* < 0.001, *p* < 0.001, respectively). During dietary treatment, group A progressively dismissed ketoprofen-based pharmacological treatment (*p* = 0.002) from baseline to t1. Furthermore, a decrease in the platelet-to-lymphocyte ratio and neutrophil-to-lymphocyte ratio at t1 in group A (*p* = 0.02) was observed compared to group B. Concluding, this study provides evidence of a positive impact of ω-3-enriched MMD regimen on the inflammatory status and MH.

**Clinical trial registration:** https://clinicaltrials.gov/ct2/show/NCT01890070, identifier: NCT01890070.

## Introduction

Morning Headache (MH) is generally associated with sleep disorders (due to stress, anxiety, insomnia), obstructive sleep apnea syndrome (OSAS), snoring, mood disorders, alcohol abuse, bruxism, and hypertension (1). The pathogenesis of MH is still uncertain (1). The International Classification of Headache Disorders (ICHD) provided the first line criteria for classifying and diagnosing migraine; MH often exhibits migraine characteristics (2). According to Ohayon, M. M (1), the main sociodemographic determinants positively related to MH are being middle-aged and unemployed or a homemaker, and being a woman. However, examining the frequency of MH in a community-based sample, Ulfberg J. et al. did not find significant differences between men and women (3). With a prevalence of approximately 1 in 13 in the general population, MH shows several different symptoms than other neurological diseases (1). Indeed, Viana et al. have described an interesting variability of clinical presentation in a small group of enrolled patients and the same patient. Symptoms recorded during this trial were: pain intensity, nausea, vomiting, photophobia, phonophobia, osmophobia, and allodynia (4). Among the various hypotheses underlying the genesis of migraine, vascular dysfunction, cortical diffusion depression, activation of the trigeminovascular pathway, and excessive pro-inflammatory and oxidative states have been indicated (5). Despite the countless familiar clinical history, the only associations between genetic polymorphisms and migraine are related to the Familiar Hemiplegic Migraine (FHM), a rare Mendelian form of migraine (6). Regarding the neurons and the intracellular signaling pathways, several studies designate a pivotal role for the Calcitonin Gene-Related Peptide (CGRP) as a mediator of migraine and a possible therapeutic target.

The pharmacological response to migraine is highly variable. Migraine attacks may respond heterogeneously to drug administration, often in the same patient (7). Multimodal therapeutic approaches are proposed to improve the quality of life to contrast a possible inflammatory state resulting from a sedentary lifestyle and increased adipose tissue (8).

There has been a growing interest in the diet therapy approach in recent years. Dietary guidelines that have been used include Ketogenic Diet (KD), low-calorie diet, Modified Atkins Diet (MAD), low-sodium diet (Dietary Approaches to Stop Hypertension, DASH), and elimination diets (9). KD and MAD, characterized by a low intake of carbohydrates (less than 50 g/day), seem to improve mitochondrial function, compensate for serotoninergic dysfunction and decrease CGRP levels, promoting neuroprotection and suppressing neuroinflammation (9). Low-calorie diets, characterized by a low intake of calories compared to the Total Daily Energy Expenditure (TDEE), can decrease migraine attacks, thanks to the reduction of body fat mass. DASH, characterized by a low sodium intake (less than 2400 mg/day), is indicated for patients with MH and vascular risk factors, such as hypertension (10). An elimination diet strategy has been introduced, considering that some foods trigger migraine attacks (11).

One of the best dietary patterns to prevent and inhibit neuroinflammation is the Mediterranean Diet (MD) (12). The MD is based on consuming several typical foods, such as whole grains, legumes, extra virgin olive oil, fish, vegetables, fruits, and nuts, which contain nutrients essential to maintaining human health. MD is rich in antioxidants, fiber, and Monounsaturated Fats (MUFAs) and an adequate balance of omega-6 (ω-6)/omega-3 (ω-3) fatty acids (FAs). It is poor in Saturated Fats (SFAs) and animal proteins (13). MD represents excellent support to therapies for chronic degenerative diseases (CDDs) (14). Moreover, Parletta et al. have recently demonstrated the correlation between MD, particularly rich in ω-3 derived from fish and olive oil, and mental health in people with depression (15). ω-3 and ω-6 FAs act as precursors of some bioactive lipid mediators, including prostaglandins, leukotrienes, resolvins, and maresins (known as oxylipins), able to regulate inflammation involved in CDDs, mental health disorder, and pain. However, ω-3 fatty acid derivatives are associated with antinociceptive and anti-inflammatory effects, while ω-6 fatty acid-derived oxylipins worsen to pain and cause migraines in several experimental models (16). Previous studies evaluating ω-3 fatty acid supplementation for migraines have been inconclusive (17). However, it has been hypothesized that diets rich in ω-3 fatty acids would increase serum 17-hydroxydocosahexaenoic acid (17-HDHA), a derivative with antinociceptive effects reducing headache-related disability (18). The dietary imbalance of high ω-6 fatty acids and low ω-3 fatty acids intake, characteristic of certain dietary patterns, such as the Western diet, may promote physical pain, and has been associated with the onset of inflammatory diseases (19). In countries adhering to a Western diet, the ω-6/ω-3 ratio is 15–17:1 (19), while it is about 10:1 (20) in Italy, a Country based on Mediterranean foods. Therefore, identifying the right ω-6/ω-3 ratio is of primary importance, rather than modulating the intake of only one of the two classes of fatty acids.

Considering this, the study aims to compare the clinical efficacy of a Modified Mediterranean Diet (MMD) with two different ω-6/ω-3 FAs ratios in adults with MH in terms of reduction of symptoms, pharmacological treatment, and body weight (21).

## Materials and methods

### Study design and subjects

The MH trial, registered on ClinicalTrials.gov (Registration Number: NCT01890070), was conducted in Clinical Nutrition and Nutrigenomics Section, of the Department of Biomedicine and Prevention of the University of Rome Tor Vergata, from May to September 2021.

The MH clinical trial has been structured as a randomized, rater-blind study, carried out on 6 weeks of dietary intervention.

Inclusion criteria were the following: a headache neurologist certification of MH; diagnosis of MH with clinical history > 6 months and with symptoms duration > 4 weeks; Caucasian males and females, aged 18–65 years old; Body Mass Index (BMI) ranged between 18.5 and 25 kg/m^2^; Nonsteroidal Anti-Inflammatory Drugs (NSAIDs) therapies; willingness to comply with study procedures, including maintaining an online daily headache diary. Exclusion criteria were: any comorbidities, drug or alcohol abuse, psychiatric pathologies, pregnancy and breastfeeding.

According to these criteria, the subjects eligible for the study underwent an online assisted medical examination at baseline and the end of dietary intervention due to restrictions during the COVID-19 lockdown. Participants recorded headaches and drug use in an online daily headache diary. Patients were asked to undergo clinical testing at an accredited clinical laboratory.

During the first visit, the enrolled patients were randomly assigned, in a 1:1 ratio, to one of two dietary interventions (A and B). A modified rater-blind study was used with only the dietitian unmasked at randomization, able to assign diet plans. All staff involved in the clinical trial was masked for group assignment. Participants did not know the nature of the other intervention.

An evaluation of nutritional status was conducted at baseline and after 6 weeks (t1) through accurate data collection (22).

At randomization, to increase diet adherence intensive dietitian counseling, a meal plan, a list of foods and food quantities to buy weekly, and 20 recipes were provided. Patients were monitored during the 6 weeks by telephone interview, performed once a week. They were asked about their general opinion on their satisfaction with the diet. To evaluate the food intake, a Dietary intake assessment (24-h recall) was performed on three non-consecutive days during the baseline phase and repeated during the weeks of the intervention phase. Adverse events were assessed. Participants were asked about the rash, tissue swelling, shortness of breath, swollen tongue, fatigue, and weight change.

The study was conducted following national and international regulations and the Declaration of Helsinki (2000). All participants were informed about the study objectives and gave consent to process data on the privacy policy. The approval of the study was obtained by the Ethics Committee of the Calabria Region Center Area Section (Register Protocol No. 146 17/05/2018).

### Anthropometrics, resting energy expenditure, laboratory tests, inflammatory risk indexes, and drug history

Body weight and height were measured using a scale and a stadiometer at home while the subject was standing wearing underwear. The data were collected to the nearest 0.1 kg and 0.1 cm, respectively. Neck, waist, and hip circumferences were measured with a flexible and non-extensible metric tape. BMI was calculated as body weight (kg)/height (m^2^) and classified according to the WHO. A Waist to Hip circumferences Ratio (WHR) was evaluated according to the clinical risk thresholds equivalent to WHR > 0.85 for women and WHR > 0.90 for men (22).

The Resting Energy Expenditure (REE) was determined using the Harris-Benedict formula (23). The TDEE was calculated by multiplying REE by the proper Physical Activity Level (PAL) (20).

The serological values examined were: Complete Blood Count (CBC), total Cholesterol (T-Chol), Low-Density Lipoproteins (LDL), High-Density Lipoprotein (HDL), and Triglyceride (Tg).

Platelet-to-Lymphocyte Ratio (PLR) and Neutrophil-to-Lymphocyte Ratio (NLR) were used as inflammation markers. NLR values indicative of low risk are < 1.6, for medium risk, ranged between 1.6 and 2.4, and for high risk is > 2.4 (24). The cut off for PLR is < 150 (25). NLR and PLR are considered inflammatory markers and useful for detecting subclinical inflammation (26).

During the online assisted medical examinations, patients were also interviewed about their drug history and posology.

### Dietary intervention and nutrient intake assessment

The MMDs were divided into five meals a day. The average caloric distribution of the meals was as follows: 15% of the total daily kcal for breakfast, 10% of the total daily kcal for morning snack, 35% of the total kcal/day for lunch, 10% of the total kcal/day for an afternoon snack, 30% of the total kcal/day for dinner.

The MMD, compared to a standard MD (27), provides the following macronutrient intake: 40–45% of total kcal/day of carbohydrates, 19–20 % of total kcal/day of proteins (>50% vegetable-derived), 35–37% of total kcal/day of lipids (in the total daily energy intake: saturated fat < 10%, and about 11% of sugars). The MMD A was designed to arise at least a 1.5:1 ω-6/ω-3 ratio; the MMD B was intended to arise a 4:1 ω-6/ω-3 ratio.

The MMDs were isocaloric with respect to daily energy requirements, according to patients TDEE.

The Mediterranean Adequacy Index (MAI) was calculated using the ratio of the caloric intake (% kcal/day) derived from carbohydrates and typical Mediterranean foods (like bread, pasta, vegetables, fruit, extra virgin olive oil, fish, and red wine) and non-typical ones (like meat, milk, and dairy products, eggs, sugar, sweets, and alcohol). MAI values are considered acceptable when the value is >5 and 100% adequate >15 (28).

The MD adherence of subjects was analyzed using the validated 14-items Mediterranean Diet Adherence Screener (MEDAS), which score ranges from 0 to 14 points (Appendix A in Supplementary material) (29).

The subject's food intake was assessed with a 3 days/week diet record, and a food frequency questionnaire was used to identify the weekly frequency of intake of different foods (30).

At the end of the clinical trial, the final database was elaborated.

The bromatological composition of the dietary intervention, the MAI and Oxygen Radical Absorbance Capacity (ORAC) (31) level of each diet was obtained using the diet analyzer software package Dietosystem^®^ (version 17.00, DS Medica SRL, Milan, Italy).

### Headache test (HIT-6) and VAS

The Headache Impact Test (HIT-6) measures the impact that headaches have on the ability to function on the job, at school, at home, and in social situations (Appendix A in Supplementary material). The HIT-6 test is a validated scoring test and constitutes one of the primary clinical endpoints for our study. This questionnaire consists of six questions, with a scale that ranges from 36 to 78 points. A higher score indicates a more significant impact of headaches on quality of life.

The Visual Analog Scale (VAS) represents a graduated scale ranging from 0 (the best possible state of health without pain) to 10 (the worst possible form of health, measured as the maximum grade of pain), on which a patient indicates their perceived level of pain.

Participants completed the HIT-6 and indicated the VAS before randomization and follow-up visits.

### Statistical analysis

The data collected before statistical evaluations were analyzed for the presence of outliners, and the Shapiro-Wilk test was performed to evaluate variables distribution. The Bartlett's or Levene's tests were used to test the variances' homogeneity. The data presented are expressed as mean, median, standard deviation, minimum and maximum values, and variance percentage (Δ%) to evaluate differences between the times. At baseline, the differences between group A and group B were assessed by the Mann–Whitney test. Wilcoxon signed-rank test was used to determine the presence of differences in the variables examined at the follow-up. The differences expressed as variance percentage (Δ%) between baseline and follow-up among different groups were assessed with the Mann-Whitney test. Linear regression analyses with the stepwise method were conducted to investigate the association between variables. Finally, a generalized linear model (GLM) was conducted to investigate the association and the future prediction between categorical variables (dependent) and continuous or categorical ones (independent). Results were significant for *p*-value < 0.05. Statistical analysis was performed using R (CRAN, Rcmdr package, vers. 2.7-1).

## Results

**Figure 1:**
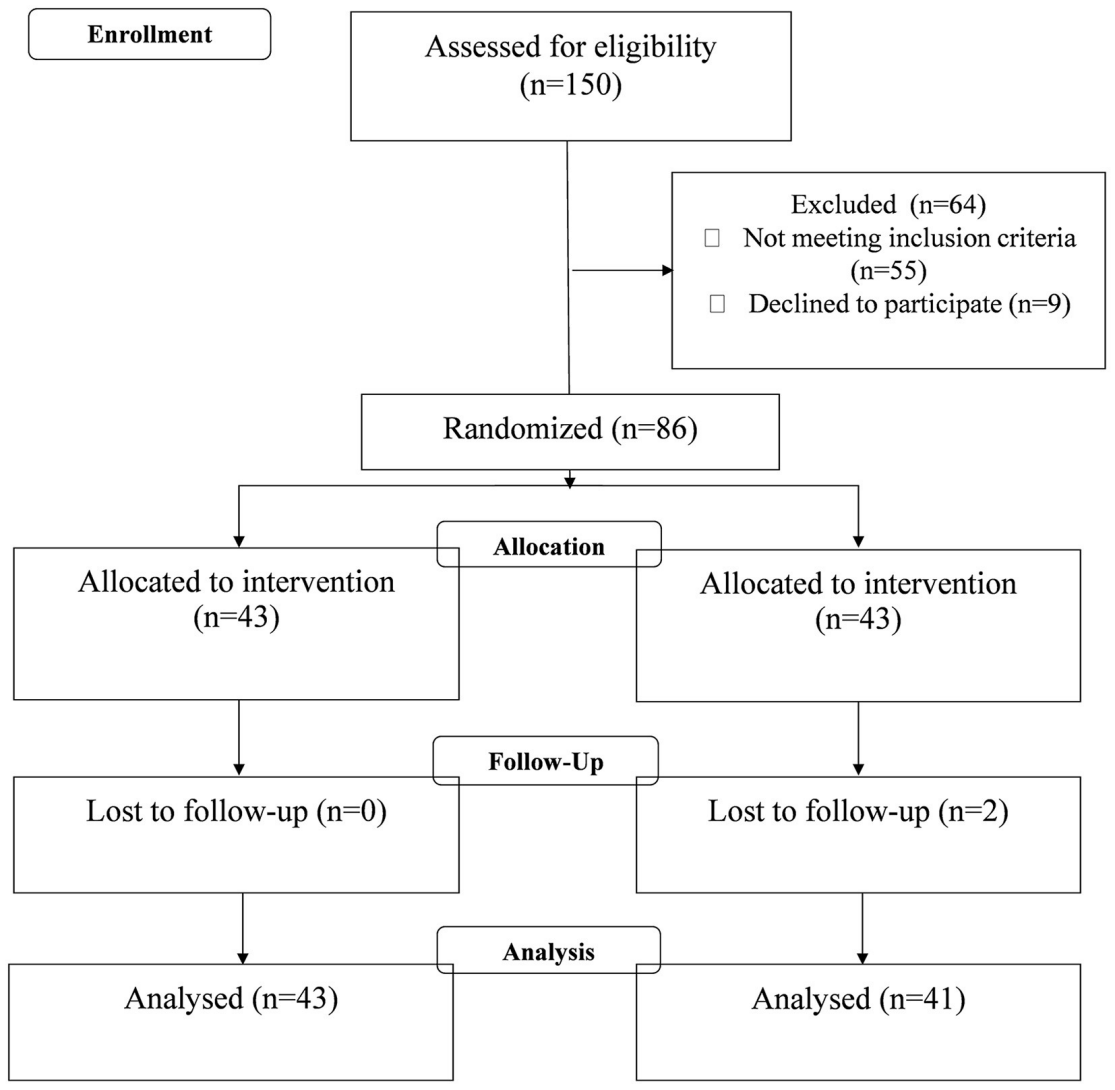
Design and subject flow through the study.

Of the 150 enrolled subjects, 95 met the inclusion criteria (63.3%); 9 declined to participate. 86 patients were randomized to two treatment groups, A and B, in a 1:1 ratio. Two patients did not complete the study. Finally, the drop-out was 11.58 %. *Eighty-four* patients, with the characteristics reported in the following Table 1, completed the 6 weeks intervention (56%). Figure 1 illustrates the design and subject flow through the study.

**Table 1:** General characteristic of the whole sample.

**Parameters**	**Values**
Participants	84
Weight (Kg)	65.4 ± 8.7
Height (cm)	168.5 ± 8.4
BMI (Kg/m^2^)	23.0 ± 1.7
Age (Years)	41.8 ± 10.0
Sex (M/F)	6/20
HIT6 score	62.6 ± 4.0
VAS score	7.4 ± 1.4

Values are expressed as mean and standard deviation (M ± SD) for continuous variables. BMI, Body Mass Index; HIT-6, Headache Impact Test; VAS, Visual Analog Scale.

At baseline, no statistical differences were observed between groups for age, height, weight, BMI, circumferences, HIT-6 score, VAS, pharmacological treatment, and serological values (data not shown).

### Dietary components

The comparison between nutrient intake at baseline and t1 (MMDS, A and B) is shown in Table 2.

**Table 2:** Nutrient intake: dietary components (macro-and micronutrients) and nutritional indexes of the usual diet (baseline) and Modified Mediterranean Diet (MMD) (respectively, for diet A and diet B).

	**Group A** ***n*** = **43 (*****F*** = **23)**			**Group B** ***n*** = **41 (*****F*** = **20)**		
**Parameters**	**Baseline**	**t1**		**Baseline**	**t1**	
	**Median** ± **[min. –max. value]**		***p*-value**	**Median** ± **[min. –max. value]**		***p*-value**
Energy (kcal)	2330.5 ± [2150.0–2570.0]	1750.0 ± [1500.0–2400.0]	0.01*	2318.0 ± [2050.0–2595.0]	1755.0 ± [1495.0–2350.0]	0.02*
Protein (% kcal)	13.6 ± [11.0–15.0]	19.7 ± [15.0–22.0]	<0.001***	13.8 ± [11.3–15.3]	20.0 ± [15.0–22.0]	<0.001***
Vegetable proteins (g)	29.0 ± [25.5–32.3]	40.6 ± [35.0–50.0]	0.003**	30.5 ± [26.3–35.1]	39.1 ± [34.0–54.0]	0.1
Animal proteins (g)	35.7 ± [15.3–60.4]	50.3 ± [16.0–73.0]	0.2	37.8 ± [16.0–61.2]	48.6 ± [15.5–72.0]	0.3
Carbohydrates (% Kcal)	43.5 ± [41.4–48.4]	48.4 ± [43.0–50.0]	0.8	45.2 ± [41.7–50.1]	42.3 ± [40.0–45.5]	0.9
Carbohydrates (g)	273.0 ± [165.0–290.0]	212.0 ± [163.0–280.0]	0.09	282.0 ± [171.0–295.0]	200.5 ± [165.0–280.0]	0.08
Sugars (g)	84.7 ± [55.0–94.0]	54.0 ± [38.0–71.0]	0.09	67.8 ± [41.0–85.3]	49.9 ± [45.0–72.0]	0.5
Total fiber (g)	20.2 ± [17.0–23.4]	38.5 ± [31.0–39.0]	<0.001***	35.5 ± [22.3–45.1]	35.3 ± [30.0–38.0]	0.9
Lipids (% kcal)	37.8 ± [35.0–40.3]	35.7 ± [31.4–37.5]	0.8	38.8 ± [36.2–41.1]	37.3 ± [32.0–38.6]	0.9
SFAs (g)	25.1 ± [13.4–22.0]	12.5 ± [7.4–18.1]	0.02*	33.4 ± [21.1–35.4]	14.5 ± [7.8–19.0]	<0.001***
ω6/ω3	5.5 ± [5.0 −8.3]	1.7 ± [1.5–1.8]	<0.001***	5.6 ± [5.1 −8.9]	4.6 ± [4.3–5.9]	0.2
ORAC (μmol)	5870.0 ± [5550.0–6340.0]	13530.0 ± [11550.0–14300.0]	0.003**	6080.0 ± [5850.0–6440.0]	11760.0 ± [11050.0–12280.0]	0.006*
MAI	1.4 ± 0.7	14.0 ± [14.0]	<0.001***	1.2 ± 1.0	14.0 ± 14.0	<0.001***
MEDAS	6.0 ± 2.0	14.0 ± [14.0]	<0.001***	6.0 ± 1.9	14.0 ± 14.0	<0.001***

Values are expressed as median, minimum, and maximum values (Median ± [min.–max. value]) for continuous variables. The pairwise Wilcoxon signed-rank test was performed to compare the dietary intake of the baseline diets and the proposed diets for both intervention groups. F, Female; SFAs, Saturated Fatty Acids; ω-6/ω-3, omega-6/omega-3 ratio; ORAC, Oxygen Radical Absorbance Capacity; MAI, Mediterranean Adequacy Index; MEDAS, Mediterranean Diet Adherence Score.

* *p* < 0.05;

** *p* < 0.005;

*** *p* < 0.001.

A statistically significant difference was observed in the ω-6/ω-3 ratio from baseline to diet A (*p* < 0.001). SFAs daily intake (expressed as grams) was decreased from baseline to diet A and diet B (*p* = 0.02, *p* < 0.001, respectively). An increase of ORAC in both A and B diets compared to the baseline diet (*p* = 0.003 and *p* = 0.006, respectively) was observed. Furthermore, a statistically significant difference was observed between baseline to diet A (*p* = 0.01) and from baseline to diet B (*p* = 0.02) for total energy daily intake (kcal). Increases in MAI (*p* < 0.001, *p* < 0.001 respectively), MEDAS (*p* < 0.001, *p* < 0.001 respectively), and protein daily intake (expressed as % of kcal: *p* < 0.001, *p* < 0.001 respectively) were observed between baseline and both diets, A and B. Furthermore, a statistically significant increase in the fiber daily intake (expressed as grams) was observed between the baseline diet and both diets, A and B (*p* < 0.001, *p* < 0.001, respectively).

Moreover, a significant difference was observed in the ω-6/ω-3 ratio, fiber intake, and ORAC between diet A and diet B (*p* = 0.001, *p* = 0.01, and *p* = 0.04, respectively). No differences for animal (g) origin proteins, carbohydrates (% kcal, g), or lipids (% kcal) were highlighted between diets at baseline and Group A and B, respectively (Table 2).

### Morning headache related – VAS and HIT-6 score

The VAS scale was administered to compare the pain sensitivity of groups A and B at baseline and after 6 weeks of dietary treatments. The pairwise Wilcoxon signed-rank test highlighted a significant decrease in VAS (*p* = 0.001) in group A from baseline to t1 (Table 3).

**Table 3:** Differences between the baseline and after 6 weeks of the MMD in each group for VAS and HIT-6 scores.

	**Group A**			**Group B**			Δ**% Baseline–t1**		
**Parameters**	**Baseline**	**t1**	***p*-value**	**Baseline**	**t1**	***p*-value**	**Group A**	**Group B**	***p*-value**
	**Median** ± **[min. –max. value]**			**Median** ± **[min. –max. value]**			**Median** ± **[min. –max. value]**		
HIT-6 score	64.0 ± [54.0–70.0]	42.0 ± [38.0–48.0]	0.001**	62.5 ± [56.0–70.0]	61.5 ± [55.0–68.0]	0.100	−33.3 ± [−25.0 to −42.0]	−10.0 ± [0 to −13.0]	<0.001***
VAS	8.0 ± [5.0–9.0]	2.0 ± [1.0–5.0]	0.001**	7.0 ± [5.0–10.0]	6.0 ± [5.0–9.0]	0.080	−73.2 ± [−37.0–−83.0]	−26.7 ± [0 to −33.0]	<0.001***
PLR	61.0 ± [50.0–69.0]	40.0 ± [35.5–45.0]	0.040*	60.0 ± [50.0–68.5]	58.0 ± [55.0–63.5]	0.300	−41.0 ± [−33.3 to −53.3]	−10.0 ± [−10.0 to −13.0]	0.070
NLR	1.0 ± [0.8–1.2]	0.4 ± [0.3–0.45]	0.020*	1.0 ± [0.7–1.2]	0.80 ± [0.5–0.9]	0.200	−63.0 ± [−60.0 to −66.6]	−19.0 ± [−10.0 to −20.0]	0.020*
Ketoprofen (mg/week)	130.0 ± [40.0–280.0]	0 ± [0–20.0]	0.002**	120.0 ± [40.0–160.0]	100.0 ± [20.0–120.0]	0.200	−100.0 ± [−50 to −100]	−17.0 ± [0 to −30.0]	0.040*

Values are expressed as median, minimum, and maximum values (Median ± [min.–max. value]) for continuous variables. Differences between both groups of dietary intervention from baseline to t1 were performed Wilcoxon signed-rank test, while Δ% Baseline−4 weeks differences between the groups were compared by Mann-Whitney test. Statistical significance was attributed as *p < 0.05; **p < 0.01; ***p < 0.001). The Shapiro-Wilk test was performed to evaluate the variable distribution. Variables are considered non-normally distributed for *p* < 0.05. HIT-6, Headache Impact Test; VAS, Visual Analog Scale.

Moreover, the MH symptoms and frequency comparison between group A and B was evaluated through the HIT-6 score and resulted in a significant decrease through the pairwise Wilcoxon rank test (*p* = 0.001) from baseline to t1 in the group A (Table 3). A significant reduction between group A and B from baseline to t1 (expressed as Δ%) was observed for HIT-6 score and VAS (*p* < 0.001 and *p* < 0.001 respectively; Table 3).

Furthermore, a linear regression analysis showed that a higher waist circumference was associated with a higher VAS (*r*^2^ = 0.86; *p* = 0.02) and abdomen circumference (*r*^2^ = 0.82, *p* = 0.01).

Using a GLM, a direct correlation between the perception of pain (linked to the VAS) and the waist circumference of patients was observed (Table 4). Interestingly, a decrease in VAS and waist circumference (*p* = 0.04) from baseline to t1 compared to group B was observed in group A.

**Table 4:** Generalized linear model (GLM) for different types of diet (i.e., group A and group B, at baseline and follow-up, respectively) on VAS and waist circumference loss (expressed as Δ% from baseline to t1) during 6 weeks of dietary treatment.

**Coefficients**	**Estimate**	**Std. Error**	***z*-value**	***p*-value**
Intercepts	−9.1263	5.2544	−1.737	0.004**
VAS (Δ%Baseline-t1)	3.4622	2.0385	2.601	0.009**
Waist circumference (Δ%Baseline-t1)	1.4481	0.9654	−1.737	0.04*
Null deviance: 30.4985 on 21 degrees of freedom				
Residual deviance: 6.1056 on 19 degrees of freedom				
AIC: 12.16				

Statistical significance was attributed as *p < 0.05; **p < 0.01; ***p < 0.001). VAS, Visual analog scale.

### Low-grade chronic-inflammation related morning headache and quality of life

Differences between inflammation indexes (i.e., PLR, NLR) of groups A and B were performed by Wilcoxon signed-rank test. A significant decrease in PLR of group A from baseline to t1 (*p* = 0.04) was observed. A significant decrease in NLR of group A from baseline to t1 was observed (*p* = 0.02) (Table 3). Furthermore, a significant decrease between groups A and B from baseline to t1 (expressed as Δ%) was observed for NLR (*p* = 0.02) (Table 3).

Pharmacological therapy for headache symptoms and the ketoprofen administration have been analyzed. During the dietary intervention, a significant reduction of ketoprofen administration was observed at t1 in group A (*p* = 0.002), while group B remained substantially unchanged (Table 3).

### Anthropometry and serological screening

At baseline, both groups A and B patients followed an unbalanced diet, characterized by a high-fat and a large amount of refined carbohydrates intake, evaluated by the food diary analysis. Isocaloric MMDs result in a weight loss in both groups (group A: *p* = 0.001; group B: *p* = 0.002) after 6 weeks of dietary intervention. A higher significant decrease was observed in group A from baseline to t1 (*p* = 0.001) compared to group B (*p* = 0.002) (Table 5). A significant decrease in the anthropometric measures (i.e., waist, abdomen, and hip circumferences) from the baseline to t1 for group A (*p* = 0.004, *p* = 0.003, and *p* = 0.001 respectively) and group B (*p* = 0.01, *p* = 0.01, and *p* = 0.02) was observed (Table 5).

**Table 5:** Comparisons from baseline to t1 for anthropometrics and serological parameters between groups A and B.

	**Group A**			**Group B**			Δ**% Baseline–t1**		
**Parameters**	**Baseline**	**t1**	***p*-value**	**Baseline**	**t1**	***p*-value**	**Group A**	**Group B**	***p*-value**
	**Median** ± **[min.–max. value]**			**Median** ± **[min. –max. value]**			**Median** ± **[min. –max. value]**		
Weight (Kg)	68.0 ± [58.0–85.0]	64.0 ± [56.0–81.0]	0.001**	62.5 ± [49.6–78.0]	61.0 ± [50.0–75.0]	0.02*	−3.0 ± [0 to −4.7]	−1.7 ± [0 to −4.6]	0.9
WC (cm)	82.0 ± [58.0–100.0]	76.5 ± [58.0–99.0]	0.004**	76.0 ± [60.0–97.0]	75.5 ± [63.0–95.0]	0.01*	−2.2 ± [0 to −5.0]	−1.0 ± [0 to −2.0]	0.20
AC (cm)	90.5 ± [67.0–109.0]	85.50 ± [66.0–106.0]	0.003**	84.0 ± [78.0–104.0]	80.0 ± [91.0–111.0]	0.01*	– 3.2 ± [0 to −6.2]	−1.0 ± [0 to −3.5]	0.20
HC (cm)	100.5 ± [90.0–107.0]	97.0 ± [89.0–106.0]	0.001**	94.0 ± [91.0–113.0]	90.5 ± [79.0–101.0]	0.02*	−1.0 ± [0 to −4.0]	−1.0 ± [0 to −3.0]	0.60
WHR	0.82 ± [0.80–0.83]	0.78 ± [0.76–0.80]	0.50	0.81 ± [0.80–0.83]	0.79 ± [0.76–0.80]	0.50	−4.9 ± [0 to −5.0]	−4.6 ± [0 to −5.0]	0.60
Tg (mg/dl)	133.0 ± [59.0–280]	100.0 ± [52.0–126.0]	0.002**	97.0 ± [57.0–117.0]	94.0 ± [57.0–117.0]	0.20	−7.8 ± [−7.0 to −59.0]	−0.3 ±[0 to −6.0]	0.50
T-Chol (mg/dl)	220.0 ± [150.0–240.0]	208.0 ± [130.0–230.0]	0.90	195.0 ± [140.5–220.0]	190.0 ± [140.0–210.5]	0.90	−2.9 ± [−2.7 to −10.0]	−5.0 ± [66.0–106.0]	0.80
LDL (mg/dl)	145.0 ± [115.5–200.0]	135.0 ± [110.5–190.0]	0.70	112.0 ± [100.5–180.0]	110.0 ± [100.0–170.5]	0.8	−2.9 ± [−2.7 to −10.0]	−2.0 ± [−2.7 to −8.0]	0.8
HDL (mg/dl)	56.5 ± [45.0–65.0]	57.5 ± [45.5–65.5]	0.8	57.0 ± [47.5–65.0]	56.5 ± [48.0–66.0]	0.7	1.8 ± [0–16.0]	−7.0 ± [−9.0 to −11.0]	0.5

Values are expressed as median, minimum, and maximum values (Median 1 [min.–max. value]) for continuous variables. Differences between both groups of dietary intervention from baseline to t1 were performed Wilcoxon signed-rank test, while 1% Baseline−4 weeks differences between the groups were compared by Mann-Whitney test. Statistical significance was attributed as *p < 0.05; **p < 0.01; ***p < 0.001. The Shapiro-Wilk test was performed to evaluate the variable distribution. Variables are considered non-normally distributed for *p* < 0.05. WC, Waist circumference; AC, Abdomen circumference; HC, Hip circumference; WHR, Waist-to-hip ratio; T-chol, Total Cholesterol levels; Tg, Triglycerides levels; LDL, low-density lipoprotein Cholesterol; HDL, high-density lipoprotein cholesterol; 6 weeks (t1).

In both groups, hematochemical tests were evaluated, showing a significant decrease in triglycerides levels from baseline to t1 in group A by performing the pairwise Wilcoxon signed-rank test (*p* = 0.002) (Table 5). On the other hand, triglycerides levels in group B remained unchanged. No other statistical differences were determined at the follow-up.

## Discussion

A balanced and healthy diet that contains an optimal ω-6/ω ratio can be helpful for migraine prevention. The dietary source of ω-3 FAs includes oily fish, such as mackerel, herrings, sardines, salmon, tuna, trout, sea brass, sea bream, linseed oils, nuts, legumes, and leafy vegetables (32). Instead, ω-6 FAs are provided by olive oil, safflower and sunflower oils, corn oil, and peanut oil (13). Regarding the molecular mechanisms, ω-3 FAs exert an inhibitory effect on activating both innate and adaptive immune systems. Furthermore, they show anti-inflammatory and antioxidant properties able to remove the Reactive Oxygen Species (ROS) directly. In particular, Eicosapentaenoic Acid (EPA) and Docosahexaenoic Acid (DHA) are enzymatically converted to Specialized pro-resolving Mediators (SPMs) known as resolvins, protectins, and maresins, and their functions can orchestrate the resolution of inflammation. These molecules can manage the balance of the intracellular redox status, supporting the immune system and mitigating the adverse effects of inflammation (33). Several families of oxylipin receptors are present in the trigeminal nerve endings and central pain processing pathways and regulate the sensitization and release of Calcitonin Gene-Related Peptide (CGRP). This implies a direct link between ω-6 and ω-3 FAs and headache pathogenesis. Since the human body cannot synthesize them, the only way to take them is through the diet (18, 34).

According to the recent scientific evidence, our study hypothesized that an MMD, with an optimal ω-6/ω-3 ratio, would modify the inflammatory status, consequently reducing the frequency and the intensity of the headache (18, 35).

Recently, Fila et al. have provided an exciting correlation between several micronutrients and oxidative stress in migraine. In this way, MMD could be the best dietary pattern for the high amount of ω-3 FAs (9, 36). MMD could interact with this inflammation pathway, blocking Nuclear Factor Kappa-Light-Chain-Enhancer of Activated B cells (NF-kB) related gene expression and the inflammation-based headache (18).

According to the American Headache Society, which assessed the evidence of migraine pharmacotherapies, antiemetic therapy (e.g., metoclopramide, prochlorperazine) alone can be used to relieve mild or moderate migraine attacks, although NSAIDs or acetaminophen are usually the drugs of choice to treat mild to moderate migraine attacks (37). If these drugs are ineffective, generally triptans or dihydroergotamine are considered. If the mild attacks get worse or if the attacks are severe from the beginning, triptans or dihydroergotamine can be used (37). When nausea is prominent, the combination of a triptan with an antiemetic at the onset of attacks is effective. Triptans are selective agonists of serotonin (5-HT) 1B and 1D receptors. They are not analgesics themselves, however they specifically block the release of neuropeptides that trigger migraine pain. Triptans are most effective when taken early in the attack. However, excessive use of these drugs can also lead to drug-abuse headaches (37). Furthermore, triptans and dihydroergotamine can cause coronary artery constriction and are therefore contraindicated in patients with coronary artery disease or uncontrolled hypertension; these drugs should be used with caution in elderly patients and in those with vascular risk factors (38, 39). Ubrogepant and rimegepant, namely also gepants, which are antagonists of the small-molecule calcitonin gene-related peptide (CGRP) receptor, are sound alternatives without any cardiovascular and gastrointestinal effect. Nevertheless, the anti-CGRP antibodies administration is not very frequent, probably due to the high cost-effectiveness (40). Finally, lasmiditan, a novel selective 5-HT 1F receptor agonist or gepant, such as ubrogepant or rimegepant, can be used when triptans or dihydroergotamine are contraindicated due to cardiovascular disorders, because, for its higher affinity for 5-HT 1F than for 1B receptors, has no cardiovascular contraindications.

**Figure 2:**
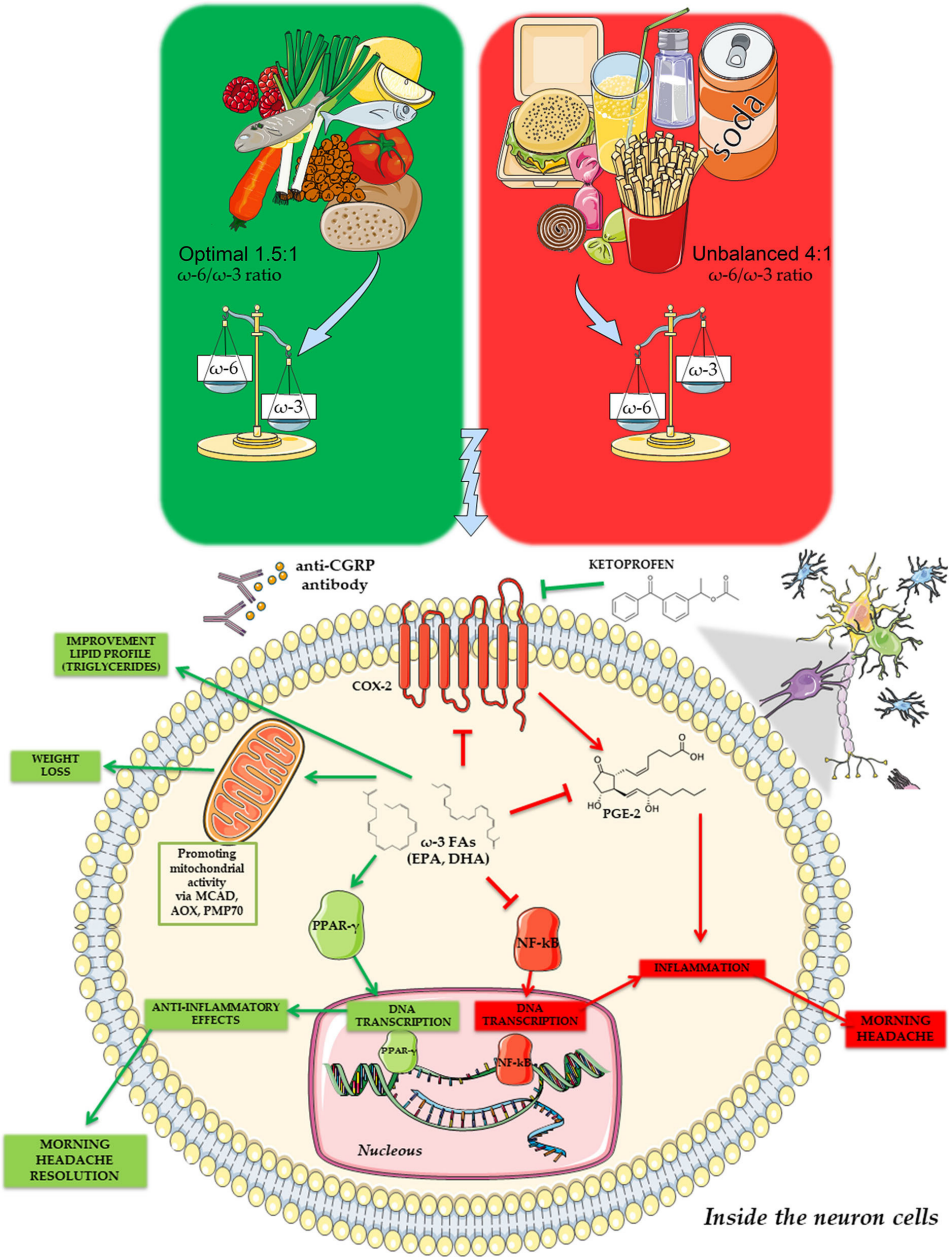
Possible signaling pathway for the connection between ω-3 and inflammation status in the morning headache development and symptoms.

NSAIDs inhibit the conversion of ω-6 arachidonic acid into the proinflammatory class of series two prostaglandins. An optimal ω-6/ω-3 ratio could mimic this mechanism, with a consequent lower need for symptomatic treatment of MH (41). According to this data, a relationship between ω-3 and the inhibition of COX-2 and NF-kB intracellular signaling pathway as the main key actors involved in the inflammation-related MH (Figure 2) has been postulated.

In the present study, a significant decrease in the inflammatory biomarkers PLR and NLR, as shown Table 3, has been observed. Therefore, the pharmacological treatment reduction could be correlated with a lower inflammatory response resulting from the proposed dietary treatment.

Several studies have shown the effect of low-fat diets in migraine prophylaxis (42). It is well known that reducing dietary fat intake for 3 months leads to a reduction in headache intensity, frequency, and drug consumption. Interestingly, these results are achieved only and exclusively with an adequate dietary intake of ω-6/ω-3 ratio (43). In fact, according to our results, individuals who follow a diet rich in ω-3 and low in ω-6 experience a greater improvement in headaches than migraineurs on a low ω-6 diet. These improvements seem to be related to the tuning of the inflammatory mediators, to the lower vasodilation, which is mostly induced by ω-6 and finally to the maintenance of physiological levels of coagulation and serotonin release by platelets, all phenomena promoted by high-fat diet, in particular by ω-6/ω-3 ratio imbalance in favor of the formers (43). Despite the shortness of the intervention and the small sample, results were encouraging. After 6 weeks of MMD, both HIT-6 score and VAS scores had a statistically significant improvement in the group A, which followed the MMD with a 1.7 ± [1.5–1.8]:1 ω-6/ω-3 ratio, with respect to group B, which followed the MMD with a 4.6 ± [4.3–5.9]:1 ω-6/ω-3 ratio. Subsequently, a reduction in frequency and intensity of MH occurs, effectively increasing the patients' quality of life. Indeed, all group A patients progressively dismissed the ketoprofen-based pharmacological treatment during the dietary treatment (44).

For the first time, evidence of a correlation between inflammatory status and morning headache development was provided. The inflammation indexes (i.e., PLR, NLR), often altered in an unbalanced diet, were significantly modified after 6 weeks of treatment with MMDs between the two groups. In particular, the PLR and NLR indexes in group A, which followed the MMD with the 1.7 ± [1.5–1.8]:1 ω-6/ω-3 ratio, are statistically lower than their values at baseline and in group B.

Moreover, according to Torres-Castillo et al. (45), a positive correlation between ω-3 and weight/circumference loss was confirmed. This is also associated with the decrease in HIT-6 score and VAS for morning headache from baseline to t1 in group A.

The association between a decrease in VAS and waist and abdomen circumferences in group A and the possible prediction for increased headache intensity with a higher waist circumference was demonstrated. Starting with these considerations, the importance of MMD and nutritional status (e.g., body weight, circumferences, BMI, dietary habits) in the morning headache prevention (9) has been highlighted. Despite the normal-ranged starting BMI of patients, an association between the waist circumference loss during dietary treatment and the VAS reduction in a generalized linear model has been observed. A strength of our study is the feasibility of MMD with respect to other diet therapies, such as elimination diets or severe restrictions (e.g., KD therapy) (10, 11). Our diet therapy plan, unlike the latter, does not prescribe substantial restrictions and deprivation of certain foods and its advantage is a varied and balanced diet consisting of a valid aid in the treatment of headaches. Another peculiarity of our study is the long-term sustainability of the proposed MMD, despite the other dietary assessments for migraine management.

Therefore, analyzing our study's characteristics and clinical outcomes, we were able to classify the 1.5:1ω-6/ω-3 ratio as the optimal for a MD, and we defined this ω-3 enriched MD as a sustainable therapeutic and preventive model for the morning headache.

The main limitation of the present study is represented by an evident difficulty in conducting medical examinations and diet interventions due to the COVID-19 pandemic restrictions. Furthermore, the small sample and the lack of inflammation markers (e.g., NF-kB, IL-6, TNF-α, C Reactive Protein – CRP) at the baseline and follow-up do not allow us to better evaluate pro-inflammatory indices, except for PLR and NLR. Still, another limitation could be the lack of an ω-6-free study group to evaluate their interaction in the inflammatory response.

Future research, it would be helpful to integrate these data in a larger sample and evaluate the body composition through a Dual-Energy X-ray Absorptiometry assay to better analyse the body fat mass. This could be helpful to better analyse the connection between pro-inflammatory status and body fat mass in morning headache development.

## Conclusion

Two personalized dietary interventions were assessed to produce biochemical changes. Our study provides, for the first time, evidence on the role of ω-3 enriched MD on MH. During ω-3 enriched MD, the migraine symptoms, frequency, and pain intensity, measured by HIT-6 and VAS tests, undergo a strong decrease.

Our results demonstrate that pain can be treated through dietary modifications, effectively leading to future novel approaches to chronic pain management. Furthermore, we observed an improvement in the quality of life, determining the reduction of ketoprofen-based pharmacological treatment, which also translates into greater patient compliance.

As a future perspective, a more detailed analysis of body composition, body fat mass, and related migraine development could provide a new personalized horizon in the coadjutant therapy for the morning headache.

## Data availability statement

The original contributions presented in the study are included in the article/Supplementary material, further inquiries can be directed to the corresponding author.

## Ethics statement

The studies involving human participants were reviewed and approved by the Ethics Committee of the Calabria Region Center Area Section (Register Protocol No. 146 17/05/2018) and conducted in accordance with the Declaration of Helsinki. The patients/participants provided their written informed consent to participate in this study.

## Author contributions

ADL and LDR: conceptualization. LDR: methodology and project administration. GB: software and formal analysis. PG and GB: validation. MM, GF, GS, and AS: investigation visualization. DT and GF: data curation. PG, MM, RC, DT, GF, GS, AS, and LDR: writing—original draft preparation. PG, ADL, RC, DT, and LDR: writing—review and editing. PG, RC, DT, AS, and LDR: supervision. All authors have read and agreed to the published version of the manuscript.

## Conflict of interest

The authors declare that the research was conducted in the absence of any commercial or financial relationships that could be construed as a potential conflict of interest.

## Publisher's note

All claims expressed in this article are solely those of the authors and do not necessarily represent those of their affiliated organizations, or those of the publisher, the editors and the reviewers. Any product that may be evaluated in this article, or claim that may be made by its manufacturer, is not guaranteed or endorsed by the publisher.

## Supplementary material

Supplementary material 1 Headache impact test – HIT-6.

Supplementary material 2 MEDAS.
