# Supplementary material for: Dietary ω-3 intake for the treatment of morning headache: A randomized controlled trial
Source: Front Neurol. 2022 Sep 20;13:987958. doi: 10.3389/fneur.2022.987958 (PMC9530603; doi:10.3389/fneur.2022.987958)
Supplement: Supplementary material 1 — Headache impact test – HIT-6. [file Data_Sheet_1.docx]

**
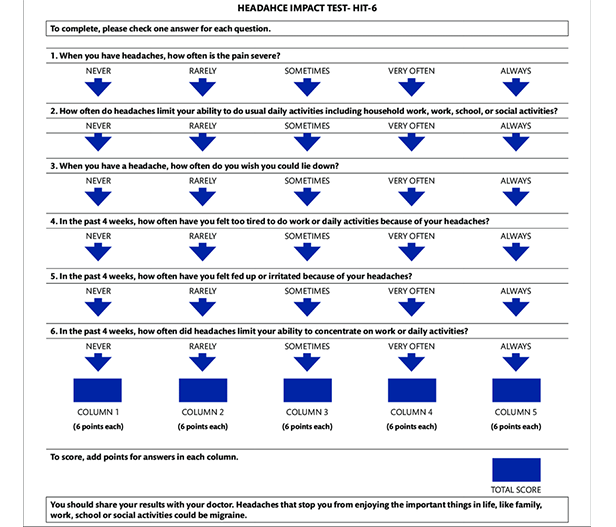
**

- - - - Scored 60 or More: Your headaches have a very severe impact on your life. You may be experiencing disabling pain and other more severe symptoms than those of other headache sufferers. Don’t let your headaches stop you from enjoying the important things in your life, like family, work, school, or social activities. Today, make an appointment to discuss your HIT-6 results and your headaches with your doctor.
      - Scores ranged from 56 – 59: Your headaches have a substantial impact on your life. As a result, you may be experiencing severe pain and other symptoms, causing you to miss some time from family, work, school, or social activities. Today, make an appointment to discuss your HIT-6 results and your headaches with your doctor.
      - The score ranged between 50 – 55: Your headaches seem to be having some impact on your life. Your headaches should not make you miss time from family, work, school, or social activities. Make sure you discuss your HIT-6 results and your headaches at your next appointment with your doctor.
      - Score 49 or Less: Your headaches seem to have little to no impact on your life. We encourage you to take HIT-6 monthly to continue to track how your headaches affect your life.
