# Supplementary material for: Dietary ω-3 intake for the treatment of morning headache: A randomized controlled trial
Source: Front Neurol. 2022 Sep 20;13:987958. doi: 10.3389/fneur.2022.987958 (PMC9530603; doi:10.3389/fneur.2022.987958)
Supplement: Supplementary material 2 — MEDAS. [file Data_Sheet_2.docx]

1. Is olive oil the main culinary fat used?

2. Are four tablespoons of olive oil used each day?

3. Are two servings (of 200 g each) of vegetables eaten each day?

4. Are three servings of fruit (of 80 g each) eaten each day?

5. Is < 1 serving (100-150 g) of red meat/hamburgers/other meat products eaten each day?

6. Was < 1 serving (12 g) of butter, margarine, or cream eaten each day?

7. Is < 1 serving (330 ml) of sweet or sugar-sweetened carbonated beverages consumed each day

8. Are three glasses (of 125 ml) of wine consumed each week?

9. Are three servings (of 150 g) of legumes consumed each week?

10. Are three servings of fish (100-150 g) or seafood (200 g) eaten each week?

11. Is < 3 servings of commercial sweets/pastries eaten each week?

12. Is one serving (of 30 g) of nuts consumed each week?

13. Is chicken, turkey, or rabbit routinely eaten instead of veal, pork, hamburger, or sausage?

14. Are pasta, vegetable, or rice dishes flavored with garlic, tomato, leek, or onion eaten ≥ twice a week?
